# Aberration hubs in protein interaction networks highlight actionable targets in cancer

**DOI:** 10.18632/oncotarget.25382

**Published:** 2018-05-18

**Authors:** Mehran Karimzadeh, Pouria Jandaghi, Andreas I. Papadakis, Sebastian Trainor, Johan Rung, Mar Gonzàlez-Porta, Ghislaine Scelo, Naveen S. Vasudev, Alvis Brazma, Sidong Huang, Rosamonde E. Banks, Mark Lathrop, Hamed S. Najafabadi, Yasser Riazalhosseini

**Affiliations:** ^1^ Department of Human Genetics, McGill University, Montreal, QC H3A 1B1, Canada; ^2^ McGill University and Genome Quebec Innovation Centre, Montreal, QC H3A 0G1, Canada; ^3^ Department of Biochemistry, The Rosalind and Morris Goodman Cancer Centre, McGill University, Montreal, QC H3G 1Y6, Canada; ^4^ Leeds Institute of Cancer and Pathology, University of Leeds, Cancer Research Building, St James's University Hospital, Leeds, LS9 7TF, UK; ^5^ European Molecular Biology Laboratory, European Bioinformatics Institute, EMBL-EBI, Wellcome Trust Genome Campus, Hinxton, CB10 1SD, UK; ^6^ International Agency for Research on Cancer (IARC), Lyon, 69008, France

**Keywords:** cancer, genomics, computational biology, systems biology, target discovery

## Abstract

Despite efforts for extensive molecular characterization of cancer patients, such as the international cancer genome consortium (ICGC) and the cancer genome atlas (TCGA), the heterogeneous nature of cancer and our limited knowledge of the contextual function of proteins have complicated the identification of targetable genes. Here, we present Aberration Hub Analysis for Cancer (AbHAC) as a novel integrative approach to pinpoint aberration hubs, i.e. individual proteins that interact extensively with genes that show aberrant mutation or expression. Our analysis of the breast cancer data of the TCGA and the renal cancer data from the ICGC shows that aberration hubs are involved in relevant cancer pathways, including factors promoting cell cycle and DNA replication in basal-like breast tumors, and Src kinase and VEGF signaling in renal carcinoma. Moreover, our analysis uncovers novel functionally relevant and actionable targets, among which we have experimentally validated abnormal splicing of spleen tyrosine kinase as a key factor for cell proliferation in renal cancer. Thus, AbHAC provides an effective strategy to uncover novel disease factors that are only identifiable by examining mutational and expression data in the context of biological networks.

## INTRODUCTION

Understanding the molecular etiology of cancer is challenging with various complexities including the multifactorial nature of the disease as well as the heterogeneity that exists at both genome and phenome levels. Advances in next-generation sequencing (NGS) have made it possible to profile multiple levels (e.g. genomic, epigenomic, and transcriptomic) of the molecular landscapes of patient samples at a high resolution. This has enabled us to identify driver abnormalities of several cancers, in particular those with a less heterogeneous molecular landscape [1–3]. However, identifying the aberrations that are functionally relevant from among the plethora of abnormal genomic patterns, particularly given the presence of many passenger events, has remained challenging for many cancers. Integrative analysis of molecular data using computational methods and prior biological knowledge has been suggested as an effective approach to find cancer driver factors [4]. However, most bioinformatics approaches that are currently used to address this issue may miss important factors that are not affected by abnormal genetic or transcriptome patterns, but are nonetheless important for development and progression of cancer, and thus, can represent novel therapeutic targets.

Integrating the protein interaction network (the “interactome”) with genomic data has emerged as a promising approach to identify novel factors that are not captured by pathway analysis [5–8]. This approach is based on the “guilt by association” principle, which assumes shared function among interacting partners in protein interaction network [9]. Interpreting protein interactions as definitive indications of shared function is an association fallacy and often false [10]; however, these interactions provide testable hypotheses which can explain the underlying biology of disease. Several such approaches have been developed that generally follow the procedure proposed by Ideker et al [11] to map p-values or z-scores obtained from differential expression or somatic mutation analyses onto the interactome in order to identify subgraphs that are highly enriched for these aberrations [12, 13]. For example, HotNet finds concentrated subnetworks of recurrent mutations by calculating an influence measure between all pairs of mutated genes, and has been successful in exploring defective interaction modules in several cancer types [13]. A more recent approach [14] uses data of individual patients independently in order to define affected subnetworks, and to distinguish driver mutated genes from passengers.

Here, we use a novel approach, Aberration Hub Analysis for Cancer (AbHAC), to identify functionally-relevant and actionable factors in cancer. AbHAC examines all individual proteins, including but not limited to hub proteins in the interaction network [15], for their direct connectivity with genes that are either significantly affected by somatic mutations or de-regulated at the mRNA level, and identifies “aberration hubs”, i.e. proteins with abnormally high interactions with genes that show aberrant mutation or expression. Therefore, AbHAC highlights candidate actionable proteins individually, and is capable of identifying factors that are not affected by genome or transcriptome aberrations themselves but are important players in cancer according to the significantly high number of deregulated proteins interacting with them.

We use several lines of evidence to show that these aberration hubs represent important cancer-related factors. First, we show that aberration hubs can distinguish different cancers and can identify relevant proteins in different subtypes of breast cancer using data from The Cancer Genome Atlas (TCGA) [16]. Next, we apply AbHAC to the International Cancer Genome Consortium (ICGC) Cancer Genomics of the Kidney (CAGEKID) data [17] for the clear cell subtype of renal cell carcinoma (ccRCC). We show that AbHAC can identify known activated molecular networks such as VEGF and Src pathways, but additionally uncovers new candidate factors. These include spleen tyrosine kinase (SYK), which we further validate as a key proliferation regulatory factor in renal cancer cells.

## RESULTS

### Implementation of AbHAC

**Figure 1:**
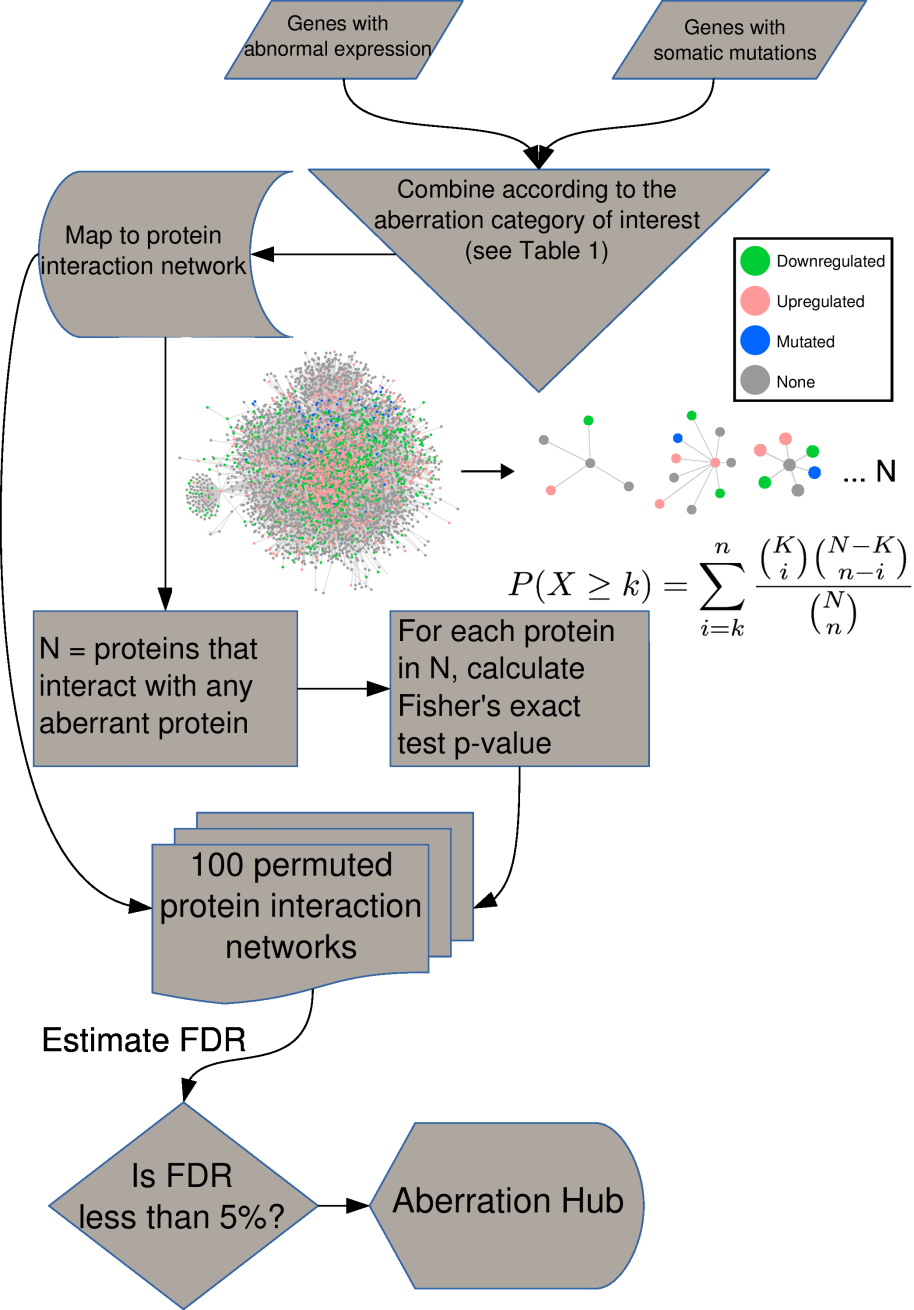
AbHAC algorithm For all the proteins in the interaction network, we assess if their local neighborhood is enriched for proteins whose coding genes are significantly mutated or/and aberrantly expressed (see Table 1 for different aberration categories). We consider each protein as an independent hypothesis, and use Fisher's exact test to evaluate over-representation of aberrant molecules among interacting partners of a given protein. We generate 100 permuted networks to correct for multiple testing.

The concept of AbHAC is to identify proteins whose local neighborhoods, constituted by their direct interacting partners, are significantly enriched for aberrant proteins (e.g. those encoded by mutated genes or translated from aberrantly expressed transcripts). AbHAC is based on the hypothesis that proteins with a significantly high number of deregulated interacting partners are likely important hubs that contribute to cancer. To implement AbHAC, we first constructed a whole human protein interaction network using the PSICQUIC [18] query system (see Methods). After excluding proteins that do not have experimentally verified annotated interactions, the interaction network included a total of 11,851 proteins with an average of 10 interactions per protein (median of 3). Then, for a particular cancer dataset, we calculated for each protein in the network the number of its direct interacting partners with and without abnormal genetic patterns (e.g. the number of interacting partners with genes identified as being significantly mutated) or abnormal expression patterns (differential expression between tumor and normal samples). As we are interested in the subset of proteins that have more abnormal partners than expected by chance, we calculated a p-value for each protein for the observed numbers of normal and abnormal partners using a one-sided Fisher's exact test (Figure 1). To correct for multiple testing, considering the complex dependencies in the protein interaction network and the resulting correlations among the p-values, we randomized the protein interaction network by permuting proteins that have similar number of interaction partners (see Methods and Supplementary Figure 1). Enrichment of various molecular aberrations can be examined by adjusting the inclusion criteria for deregulated partners. For example, we can focus uniquely on proteins whose direct interacting neighborhoods are enriched in genes deregulated at either RNA level (up- or down-regulated genes) or DNA level (significantly mutated genes), or we can undertake an integrated analysis to look for enrichment of aberrations in both RNA and DNA. Table 1 presents further examples of different aberration category queries.

**Table 1:** Definition of the aberration categories

Aberration category	Interacting partners are enriched in	Proteins enter the analysis if they have following interacting partners
**UP**	Upregulated genes	At least one upregulated
**DOWN**	Downregulated genes	At least one downregulated
**MUT**	Mutated genes	At least one mutated
**MUT.UP**	Upregulated or mutated genes	At least one mutated and one upregulated
**MUT.DOWN**	Downregulated or mutated genes	At least one mutated and one downregulated
**DE**	Upregulated or downregulated genes	At least one differentially expressed
**MUT.DE**	Differentially expressed or mutated genes	At least one mutated and one differentially expressed

### Aberration hubs are characteristics of tumor types

**Figure 2:**
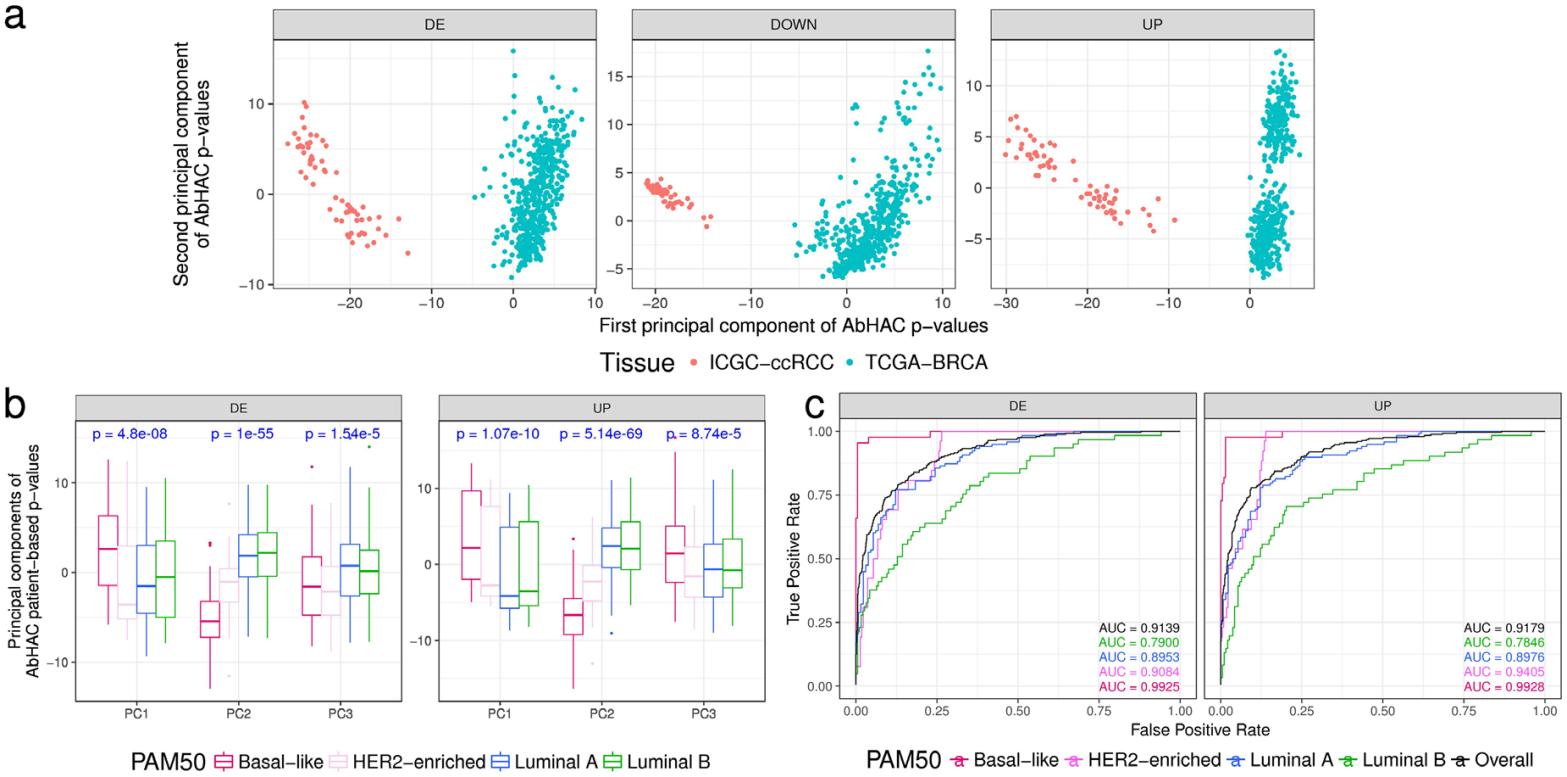
Aberration hubs distinguish between different tumor types and subtypes **(a)** Principal component analysis on AbHAC p-value matrices of TCGA breast cancer samples and ICGC renal cell carcinomas differentiates them based on tumor tissue of origin. First two principal components of AbHAC p-values for proteins enriched with differentially expressed (DE), downregulated (Down) or upregulated (UP) genes (in tumors relative to normal) among their direct interacting partners are shown. **(b)** Principal component analysis on AbHAC p-value matrices of TCGA breast cancers. For each patient, differentially expressed genes are identified as the 5 percent highest and lowest values when normalized to average values of non-tumor samples. These genes are then used for AbHAC analysis. P-values are calculated by ANOVA test. First three principal components differ among PAM50 subtypes of breast cancer. **(c)** The support vector machine classifier was trained on half of the breast tumors by grid search and 4-fold cross validation. The parameters identified by this approach were then applied on the other half of the breast tumors to predict their subtypes. The area under the curve (AUC) analyses show high sensitivity and specificity of AbHAC to predict subtype of breast cancer.

To test whether the aberration hubs are associated with clinical or phenotypic variations, we applied AbHAC to TCGA breast cancer [16] and the ICGC clear cell renal cell carcinomas (ccRCC) [17] datasets (Supplementary Tables 1-8), and investigated the distribution of aberration hubs across these samples. We calculated AbHAC p-values for all proteins in patients with breast or renal cancer (see Methods for details), and performed principal component analysis (PCA) using these p-values. This analysis showed that “aberration hubs” are different between breast cancer and ccRCC, and can be used to clearly separate patients with different cancers (Figure 2a). To confirm that this observation was not due to differences in the tissue of origin (kidney vs. breast) and rather represent cancer-associated effects, we further examined clustering of breast cancer samples using AbHAC p-values by PCA. This analysis revealed that the first three principal components differentiating between breast cancers are significantly associated with known PAM50 subtypes of breast cancer (ANOVA p < 10^-5^), confirming that the main diversity in AbHAC p-values is related to the subtype-specific differences (Figure 2b). Following this observation, we used a support vector classifier trained on half of the samples to predict the PAM50 subtype of remaining samples. This classifier achieved very high specificity and sensitivity in one-versus-all classification (see Methods and Figure 2c). Therefore, these results showed that cancer aberration hubs are not random and can provide clinical and biologically-relevant information.

### Aberration hubs in breast cancer

Breast cancer is a heterogeneous disorder with different subtypes, each characterized by distinct molecular alterations and clinical behavior [19]. The TCGA datasets provide comprehensive molecular landscapes of intrinsic subtypes as defined by PAM50 classifier [20]. Using these datasets (Supplementary Tables 1-6), we applied our statistical approach to the data from the PAM50 subtypes of breast cancer to identify proteins that might be relevant for each subtype (see Methods for details). Our analysis identified 74 aberration hubs using different aberration categories in different PAM50 classes (False discovery rate (FDR) < 0.05; Supplementary Table 9). To verify if any of the 74 proteins had previously been associated with breast cancer, we conducted a literature review using MeSHOP [21] with “Breast Neoplasms” as the MeSH term to count how often the proteins are reported to be implicated in breast cancer. We found a total of 1294 reported proteins of which 39 were in our list. This represents 4.7-fold enrichment of breast cancer-associated proteins among the identified aberration hubs compared to the background of other proteins in the interaction network (*p* = 6.9 × 10^-11^, Fisher's exact test; Supplementary Table 9), supporting the efficacy of AbHAC to identify relevant molecules in a given cancer dataset.

We further questioned if genes encoding any of these aberration hubs are among significantly mutated or abnormally expressed genes in breast cancer according to TCGA datasets. We observed that only one of these 74 molecules (CDKN1B) is significantly mutated in breast cancers, and that 50 of them are not differentially expressed at mRNA level in tumors (all together or at subtype-level) when compared to non-tumor control samples (Supplementary Table 9). In addition, only 15 of the 39 aberration hubs, which were connected to breast cancer based on previous literature, have significant differential expression at mRNA level or a mutation. This further illustrates that AbHAC is capable of identifying relevant factors that cannot be identified directly by interrogating mutational or expression patterns of genes.

Our further analysis showed that 53 of the 74 breast cancer aberration hubs are specific to one of the PAM50 subtypes in a given aberration category (Table 2, Supplementary Table 9 and Supplementary Figure 2). Pathway analysis of these proteins, using KEGG datasets, revealed that aberration hubs found in luminal breast tumors are enriched in PI3K-Akt pathway and FOXA1 regulatory network (Supplementary Table 10), in line with a high prevalence of PIK3CA and FOXA1 mutations in this subtype [22, 23]. In addition, hubs of basal-like subtype are enriched in DNA replication and cell cycle pathways (FDR < 8.75 × 10-5), which confirms previous reports on importance of these pathways in basal-like tumors [24]. Specifically, we observed several components of “origin recognition complex” (ORC) including ORC1, 2, 3, 5 and 6, and “mini-chromosome maintenance” (MCM) such as MCM3, 6 and 7, which are important factors for initiation of genome replication, among aberration hubs of basal-like tumors (Supplementary Table 10).

**Table 2:** Aberration hubs identified specific to a PAM50 subtype of breast cancer in a given aberration category by AbHAC (FDR < 0.05)

PAM50 subtype	Molecules with supporting literature in the same PAM50 subtype	Potentially novel relevant factors
**Basal-Like**	CDC45, MCM7, AURKA, PCNA, CHEK1, TFDP1	HIST1H4A, MCM3, MCM6, MCMBP, ORC6, CDC6, XRCC6, ORC2, ORC1, ORC5, WRN, NEK6, MAD2L1BP, EIF6, XRCC5, OSM, ZNF652, MYBPC2, AIRE, CHD1L, HDGF, SNW1
**HER2-enriched**	CXCR3, RACGAP1	ORC3, TONSL, COL1A2, CEACAM6, CCR3, KRT32, SEZ6L2, DPP8
**Luminal A**	HSPB8	OSM, COL5A1, PDE4DIP, EGFR, ECM1, COL5A2
**Luminal B**		ANAPC4, OPRK1, GOPC, CDC27, CDKN1B, S100A9, BRCA1, STK4, CDK3

List of all aberration hubs identified in breast cancer, their associated aberration categories, and references to the literature are presented in Supplementary Table 9.

Among the 74 aberration hubs we identified in TCGA breast cancer datasets, 45 were not affected by somatic mutations or significant changes at mRNA levels in any of the PAM50 subtypes. We hypothesized that at least some of these factors may be deregulated at translational or post-translational levels. Therefore, we investigated the status of these aberration hubs at protein level using the TCGA proteome and phosphoproteome data for breast cancer [25]. We used t-test to compare normalized expression level of each peptide or phosphopeptide in one PAM50 subtype against other PAM50 subtypes. We observed that 5 out of these 45 aberration hubs exhibit differential expression patterns at protein level (for specific phosphopeptides) in the same breast cancer subtype in which they had been identified by AbHAC as compared to tumors of other subtypes (Supplementary Table 11). These include ZNF652, XRCC5, EIF6, and HIST1H4A in basal-like, and CDC5L in luminal B tumors. These findings show that AbHAC is able to identify molecules that may specifically be implicated in a particular subtype of cancer and suggest that novel molecules identified through AbHAC approach may be functionally relevant in particular intrinsic subtypes of breast cancer.

### Aberration hubs in renal cancer

**Figure 3:**
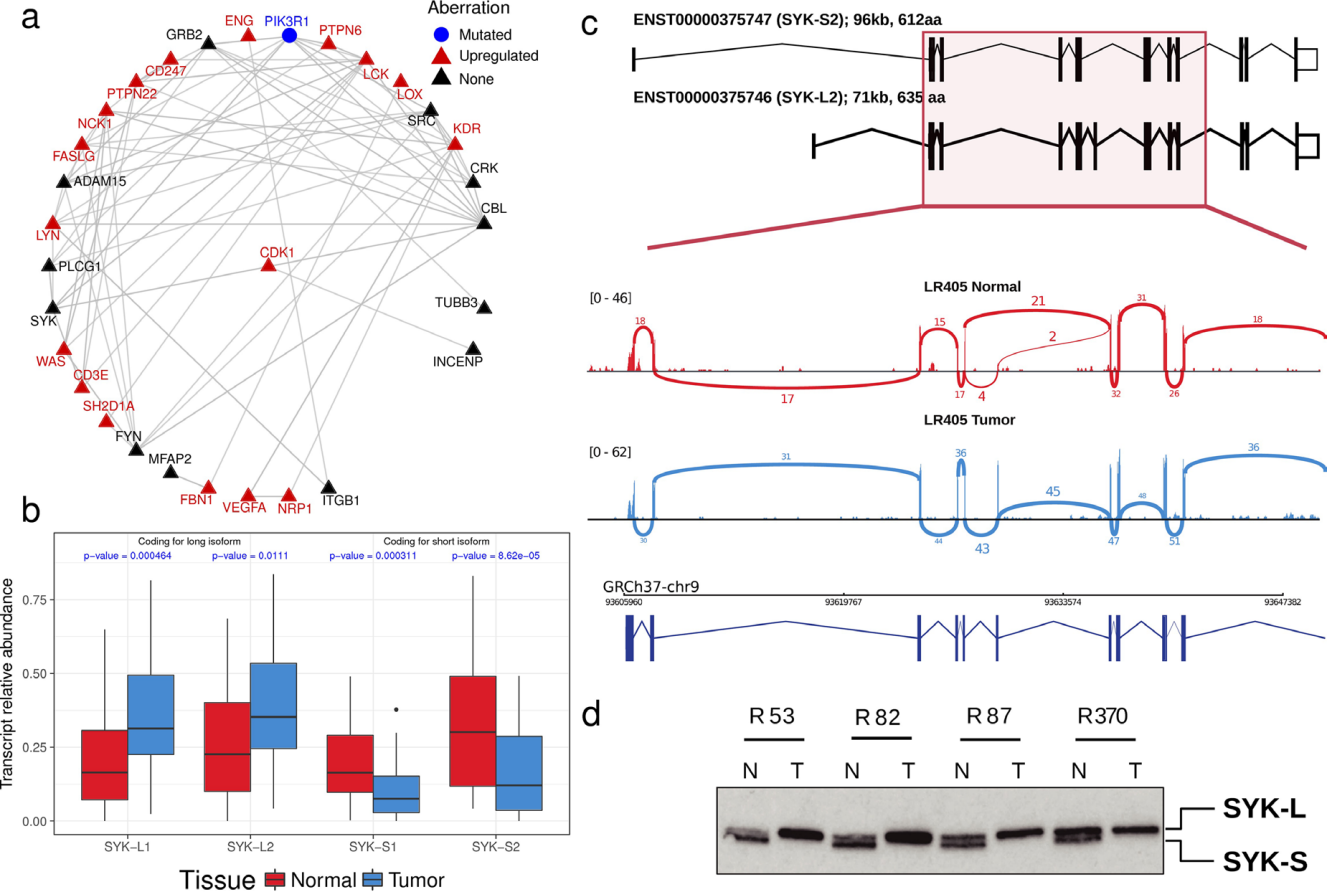
SYK, an aberration hub in ccRCC, is not affected by differential gene expression but by abnormal splicing **(a)** A core of inter-connected aberration hubs that are enriched with up-regulated genes in their interacting neighborhood in renal cancers are shown. Each gray line indicates a direct interaction. Red and black colors highlight aberration hubs that are upregulated or not differentially expressed at mRNA level in tumors as compared to normal samples, respectively. PI3KR1, represented by a blue circle, is the only mutated aberration hub. All aberration hubs identified in ccRCC by AbHAC are shown in Supplementary Figure 3. **(b)** SYK is an aberration hub that was not differentially expressed at the mRNA level. Further investigation revealed an abnormal splicing pattern between normal and tumor samples involving four SYK transcripts (ENST00000375751: SYK-S1; ENST00000375747: SYK-S2; ENST00000375754: SYK-L1; ENST00000375746: STK-L2) (see details in Supplementary Figure 4). P-values were calculated using Mann-Whitney U test. **(c)** As an example, the status of SYK spliced variants is shown for patient L405 by sashimi plot. The predominance of SYK-L2, coding for the long isoform, in tumor (denoted by blue color) compared to normal renal tissue (shown in red color) is shown. **(d)** Western blot analysis of additional sample pairs showing that the longer isoform of SYK is abundant in RCC samples (T) as compared to patient-matched normal kidney tissue (N).

**Figure 4:**
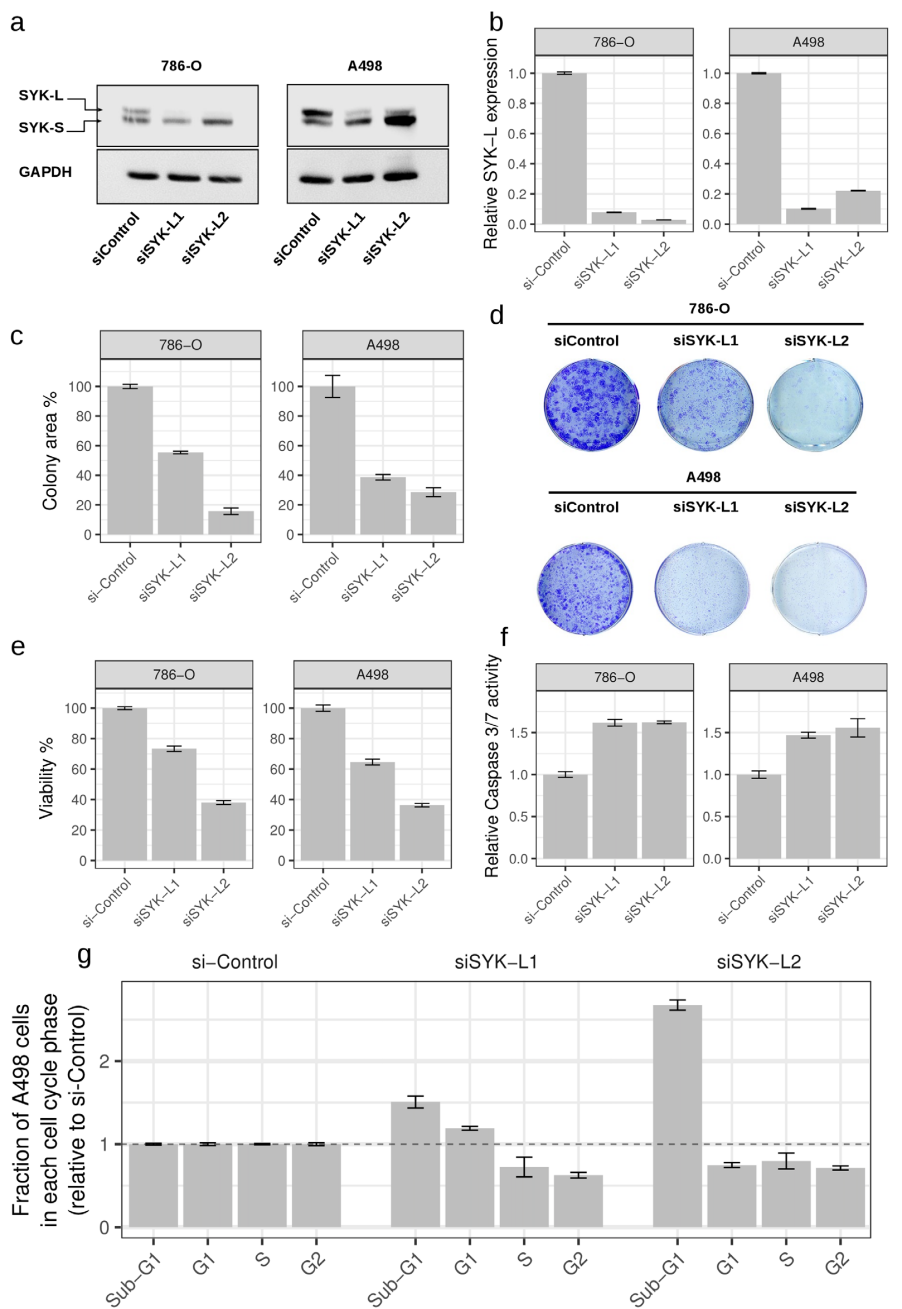
Inhibition of long isoform of SYK impairs proliferation of renal cancer cells Silencing of SYK long isoform through RNAi (siSYK-L1 and siSYK-L2) in renal cancer cell lines 786-O and A498 was confirmed by western blot **(a)** and qRT-PCR **(b)** analyses. **(c)** SYK-L knockdown reduces the colony-forming ability of 786-O and A498 cells as compared to the negative control (siControl) (n=3). **(d)** Representative images of colony formation assays are shown. Cell viability **(e)** and Caspase 3/7 activity **(f)** after knockdown of long isoform of SYK. Values are normalized to si-Control (non-targeting siRNA). **(g)** Changes in cell cycle distribution upon knockdown of long isoform of SYK in A498 cells. Whereas both SYK isoforms are detected in 786-O cells, the long isoform is predominantly detected in A498 cells and suppressed by RNAi supporting the functional relevance of this isoform.

We applied the same analysis to ccRCC using information about somatic non-silent mutations and abnormal gene expression patterns that have been recently identified through the CAGEKID program [17] (Supplementary Tables 7-8; see Methods for details). AbHAC analysis of these datasets identified 47 aberration hubs in renal tumors (FDR < 0.05; Supplementary Table 12). We noticed that, although identified independently, several of these aberration hubs were connected together through protein-protein interactions and formed a protein network that included several known cancer driver factors such as MYC, EGFR, PIK3C2B, CDK1, and KDR (Supplementary Figure 3). Further analysis of this network revealed 25 inter-connected aberration hubs that were enriched in up-regulated genes among their interacting partners. (Figure 3a, Table 3). This core was composed of several proteins with angiogenic functions in renal cancer including VEGFA, NRP1 and KDR (VEGF receptor-2), the latter of which may have predictive value in ccRCC [26]. Src kinase signaling members were also predominant in the core, including SRC, LCK, LYN, FYN and PTPN6, among which LYN has been causally implicated in renal cancer [27, 28]. In addition to interacting extensively with deregulated genes, the mRNA of many of these molecules is over-expressed in tumors as compared to normal renal tissue. This is in line with the oncogenic activities reported for them (Table 3). Notably, some proteins included in this core had not been directly reported in renal cancer (proteins without a PMID in Table 3). Among these potentially new relevant proteins, spleen tyrosine kinase (SYK) was particularly attractive, because it is a tyrosine protein kinase which has not been identified as being differentially expressed at mRNA level between tumor and control samples whereas it was identified as an aberration hub in four aberration categories (Table 3). We re-examined the RNA-Seq data of ccRCC and control samples for *SYK* transcripts, and found that while *SYK* was not differentially expressed, it was alternatively spliced in tumors as compared to non-tumor samples in 24 out of 44 (54.5%) studied patients. This was due to an aberrant splicing pattern introducing a new exon to SYK transcripts resulting in a longer isoform of the protein in tumor samples (SYK-L with 635 amino acids coded by transcripts ENST00000375754 and ENST00000375746) compared to a shorter isoform (SYK-S with 612 amino acids coded by transcripts ENST00000375751 and ENST00000375747) (Figure 3c and Supplementary Figure 4). We observed that the longer isoform of SYK was significantly more abundant in tumors as compared to normal samples (*p* < 0.008, Figure 3b and Supplementary Figure 4). We further assessed the relative abundance of the two SYK isoforms by immunoblotting in matched tumor and normal samples from four additional patients. This analysis revealed that while both isoforms were present in normal tissue specimens, only the long isoform was detected in tumors (Figure 3d). Interestingly, a similar pattern has recently been reported in ovarian cancer, where abnormal splicing of SYK supports cancer cell proliferation and survival [29]. Therefore, we used RNAi to examine whether modulation of long isoform of SYK influences the proliferation of renal cancer cells. Silencing of the SYK long isoform by two independent specific siRNAs substantially reduced proliferation of renal cancer cells in 786-O and A498 cell lines as measured by colony-formation and viability assays (Figure 4a-4e). Furthermore, suppression of SYK long isoform induced activation of apoptosis as examined by caspase assay and flow cytometry analysis (Figure 4f-4g). These findings showed that long isoform of SYK plays an important role in the proliferation and survival of renal cancer cells, highlighting the power of the AbHAC approach to identify functionally relevant factors in cancer datasets.

**Table 3:** Interconnected aberration hubs that were enriched with up-regulated genes among their interacting partners in ccRCC (FDR < 0.05)

	Uniprot	HGNC	Aberration Category^*^	Examples for supporting literature (PMID)	Status of gene expression (Tumor/Normal)
**Molecules with supporting literature**	P22681	CBL	UP & MUT.UP	21949687	NA
	P07766	CD3E	DE & UP & MUT.UP	9796963	Up-regulated
	P48023	FASLG	DE & UP & MUT.UP & MUT.DE	10353760	Up-regulated
	P06241	FYN	UP & MUT.UP	22814579	NA
	P62993	GRB2	MUT.UP	PMC2737331	NA
	P05556	ITGB1	UP	23499501	NA
	P35968	KDR	DE & UP & MUT.UP	24786599	Up-regulated
	P06239	LCK	DE & UP & MUT.UP	9796963	Up-regulated
	P07948	LYN	DE & UP & MUT.UP	22814579	Up-regulated
	O14786	NRP1	UP	18974107	Up-regulated
	P27986	PIK3R1	MUT.UP	PMC4355729	NA
	P12931	SRC	UP & MUT.UP	22814579	NA
	Q13509	TUBB3	UP	25527909	NA
	P15692	VEGFA	UP & MUT.UP	15793222	Up-regulated
**Potentially novel relevant factors**	Q13444	ADAM15	UP & MUT.UP	NA	NA
	P20963	CD247	UP	NA	Up-regulated
	P46108	CRK	MUT.UP & MUT.DE	NA	NA
	P17813	ENG	UP	NA	Up-regulated
	P16333	NCK1	MUT.UP	NA	Up-regulated
	P19174	PLCG1	MUT.UP & MUT.DE	NA	NA
	Q9Y2R2	PTPN22	UP	NA	Up-regulated
	P29350	PTPN6	DE & UP & MUT.UP & MUT.DE	NA	Up-regulated
	O60880	SH2D1A	UP	NA	Up-regulated
	P43405	SYK	DE & UP & MUT.UP & MUT.DE	NA	NA
	P42768	WAS	DE & UP & MUT.UP	NA	Up-regulated

^*^In the aberration categories, “&” refers to independent aberration categories, and “.” symbol denotes an integrative analysis including two classes of aberrations. See Table 1 for the definitions of the aberration categories.

Examples of supporting literature and status of mRNA expression are shown for each aberration hub. Definition of each aberration category is provided in Table 1.

## DISCUSSION

In the present study, we undertook a novel integrative approach to identify proteins whose interacting partners are significantly affected by abnormal molecular patterns. This was based on our hypothesis that enrichment of molecular aberrations in the local interacting neighborhood of proteins can be associated with phenotypic variations (similar to that observed for deregulation at gene expression level [30, 31]), and can therefore help identify factors that are relevant to a specific phenotype (e.g. cancer or a specific subtype of cancer). Two possible scenarios can explain function of a direct interaction between two proteins: (i) one protein modulates activity of the other protein (where direction of edges matters); (ii) both proteins form a complex to assert a function (un-directional interaction)[32]. Since our hypothesis does not prefer any of the abovementioned scenarios over the other, we assumed all protein interactions are undirected. This assumption helped expanding our analysis to include interactions for which the directionality is not known. We further validated the relevance of the identified factors by literature review and by functional experiments.

Unlike other approaches to protein network analysis, AbHAC analyzes the neighborhood of a given protein as a distinct variable independent from the rest of the interactome, and regardless of mutational or transcriptional patterns of its gene. This feature allows pinpointing “individual” proteins rather than interaction modules that may be involved in a disease. Furthermore, this feature gives AbHAC the ability to identify factors that may not be mutated or differentially expressed, but may be functionally relevant in cancer through other mechanisms. As a proof of principle, we demonstrated the functionality of this approach by showing that enrichment of abnormally expressed or mutated genes in interacting neighborhoods of proteins can classify tumors based on their pathological differences (comparison between breast and renal cancers, and between PAM50 subtypes of breast cancer). To identify proteins whose interacting neighborhoods are deregulated in a certain group of tumors, we performed Fisher's exact test and corrected for multiple testing by randomizing protein interaction networks through permuting proteins that have similar numbers of interacting partners. This permutation step provides a statistical confidence for the data generated by AbHAC by accounting for complex dependencies of any input dataset, and thereby distinguishes our method from the existing ones. For example, the DIAMOnD algorithm [33] follows a similar procedure to connect known disease genes in the protein interaction network by identifying disease modules; however, it lacks a statistical component to assign the level of significance for each observation, and to measure the false discovery rate associated with each analysis. We analyzed the same interactome and genesets (aberrantly expressed or mutated) with both AbHAC and DIAMOnD. For AbHAC we used FDR < 0.05 to identify aberration hubs, and for DIAMOnD we chose the first 100 top hits, which is larger than the maximum number of significant aberration hubs identified by AbHAC in any of our analyses. While the number of targets identified by DIAMOnD was always larger than that identified by AbHAC, there were several cases of targets only identified by AbHAC and not by DIAMOnD in this controlled comparison. For example, 29 out of 65 AbHAC targets (in 3 aberration categories of UP, DOWN or DE) were unique to AbHAC in the specific comparison of breast cancer subtypes, and 12 of these hubs had a breast cancer literature according to MeSHOP. Similarly, for renal carcinoma, 26 out of the 39 targets identified by AbHAC (in 3 aberration categories) were not among the list of DIAMOnD results (Supplementary Figure 5). VEGFA, KDR, LYN, CD3E and LOX are among those factors that are not identified by DIAMOnD but have a renal cell carcinoma associated literature support (Supplementary Table 13).

The correction for multiple testing provided a high confidence for the data generated by AbHAC. For example, only four proteins (INCENP, MIS12, CDK1, COL5A1) were common in the list of aberration hubs identified through the same analysis applied to breast cancer (with total of 74 aberration hubs) and renal carcinoma datasets (with total of 47 proteins aberration hubs), indicating that AbHAC is capable of finding proteins that are specifically deregulated in a given cancer. The above molecules identified in both breast and renal cancers have already been implicated in multiple cancers based on previous literature. For example, CDK1 is shown to be an inhibitor of FOXO1, which is known as a common tumor suppressor in different types of human cancer [34]. Accordingly, the additional factors identified in this study may present novel relevant players in breast cancer. Our results showed that several proteins with key roles in the initiation of genome replication are significant aberration hubs of basal-like tumors, shedding light on the underlying molecular mechanisms of this aggressive tumor entity. Importantly, the careful selection of genes as input for AbHAC analysis assists in the identification of functional-relevant aberration hubs. Applying analytical methods such as MuSiC [35] or MutSigCV [36], which identify significantly mutated genes, to cancer sequencing data is an appropriate approach to exclude genes affected with passenger alterations from the input list for AbHAC analysis.

To assess the reproducibility of AbHAC results, we applied this method to an additional independent genomic datasets of renal (TCGA) cancer and compared the results to our preceding findings in CAGEKID datasets. This analysis showed a strong correlation (Pearson R > 0.7, *p* < 10^-16^) between results of AbHAC analysis of these datasets (Supplementary Figure 6), suggesting that AbHAC results are not database-specific.

Furthermore, we systematically assessed the extent of possible dependency between the deregulated neighborhood of an aberration hub and deregulation of the hub itself. We compared the AbHAC p-value of proteins and the differential gene expression level of their corresponding genes in the above-mentioned datasets. We did not observe a significant correlation between AbHAC p-value and the extent of deregulation at mRNA level for a given molecule (Supplementary Figure 7). Overall, the AbHAC p-values are not significantly influenced by expression levels of the corresponding genes. In other words, aberration hubs are not necessarily affected by extreme abnormal gene expression themselves. These findings suggest that the analysis of deregulated neighborhoods among interacting partners of proteins can lead to the identification of factors that are not captured by the analysis of mRNA expression levels alone, and may therefore uncover new relevant molecules and pathways. This is exemplified by the identification of SYK in renal cancer; while AbHAC predicted SYK as a novel relevant factor, SYK had not been identified as an up-regulated gene in ccRCCs. Although we had previously identified it as being affected by abnormal splicing patterns in ccRCC [17], it was not among the top-ranked genes. Nevertheless, AbHAC highlighted SYK as a strong aberration hub based on the enrichment of abnormal genomic patterns in its direct interacting neighborhood. This example highlights the efficacy of AbHAC to identify factors that are functionally relevant in the large list of genes affected by abnormal molecular patterns in tumors. Furthermore, our findings revealed an oncogenic core signaling which is activated in tumors. Molecules involved in this network are members of VEGF signaling, the main driver of angiogenesis, which is a known target for the development of therapies against ccRCC. Resistance to anti-VEGF agents, however, often develops in patients with metastatic RCC, limiting treatment efficacy [37]. Our findings uncover novel affected factors interacting with members of this signaling pathway, which may serve as alternative targets for future drug development strategies. Interestingly, SYK has been reported as an activator of the VEGF pathway by phosphorylating VEGFR-2 receptor [38], which was also in the network that we identified in the current study. Furthermore, several members of the Src kinase family were present in this network. According to the close cross-talk between VEGF receptor and Src signaling [39], inhibition of aberrantly regulated proteins of the Src pathway may provide an additional route to control of tumor angiogenesis. Notably, Src signaling is also involved in the regulation of PI3K-Akt-mTOR pathway, which has been recognized as a relevant therapeutic target for ccRCC [40].

Collectively, our results from analyzing two cancer datasets highlighted proteins whose relevance to the studied cancers is supported by previous literature, and provided a list of potentially novel relevant factors for further investigation in future studies. Similarly, AbHAC can be applied to analyze molecular aberrations of other cancers, and other diseases in general. Likewise, the method can be used to study proteome profiles or combinations of different layers of genome and proteome information as well. As such, AbHAC and similar methods can support a systems level understanding of cancer and other complex disorders.

## MATERIALS AND METHODS

### Software packages

CRAN project R software [41] has been used for most of the analysis presented here. Some of the analysis and figures have used the following CRAN and Bioconductor packages; ggplot2 [42], limma [43], edgeR [44], PSICQUIC [18] and Uniprot.ws [45]. Figures of interaction networks were generated using cytoscape [46].

### Protein interaction network

Using PSICQUIC [18] and UniProt.ws Bioconductor packages, we acquired the protein interaction network of all the Uniprot accessions from the following databases for *Homo sapiens*: DIP, InnateDB, IntAct, MatrixDB, MINT, I2D-IMEx, InnateDB-IMEx, MolCon and BindingDB. We filtered out interactions that had been reported only based on colocalization experiments. This left us with 11,851 proteins in total that have an average of 10 interaction partners (median of 3). All duplicated interaction partners were removed in this undirected protein interaction network.

### Datasets

For the breast cancer analysis, we used the publicly available TCGA breast cancer datasets of somatic mutations and microarray expression profiles for 497 patients grouped into the 4 major PAM50 subgroups (basal-like, HER2-enriched, luminal A and luminal B)[20]. We did not include the normal-like subtype because of its significantly lower number of samples compared to other subtypes. Original PAM50 class of these patients was adopted from Supplementary Table 1 of the TCGA paper [16]. We only included mutational data for the 54 genes reported as being significantly mutated in supplementary information of the TCGA paper [22] in our analysis (Supplementary Table 1).

For the renal cancer analysis we used CAGEKID datasets [17] generated from genome-sequencing of 94 patients and RNA-Seq of 61 tumors (45 with matched normal kidney tissue control samples). Somatic mutations were included which affected 583 genes that were either recurrently mutated in ccRCCs or were identified as significantly mutated genes as reported in Supplementary data 5 of the original CAGEKID study [17] (Supplementary Table 7).

### Differential gene expression analysis

For mRNA differential expression analysis, we first converted gene symbol IDs to Uniprot accession IDs and only kept those that had any level of information in the interactome. To identify differentially expressed genes between groups of samples (all tumors or a class of tumors compared to non-tumor samples) we used limma [47] package with TREAT [48] for differential expression analysis of TCGA breast cancer normalized Agilent microarray data (level 3). The lfc parameter was set to 1, with Benjamini Hochberg (BH) FDR [49] cutoff of 0.05 in order to identify genes that are significantly differentially expressed in tumors with a minimum of 2-fold change difference compared to 22 nontumor breast tissue samples (Supplementary Table 2). This procedure was also performed for each PAM50 group of patients (except Normal-Like tumors as discussed above in the Datasets) independently (Supplementary Tables 3-6). To define genes differentially expressed in a given patient, we calculated a log2FC by dividing the expression of each gene in the patient's tumor by the average of expression values for that gene across nontumor control samples. For each patient, we considered the top 5% of genes for each direction (down or up) as differentially expressed. Differential gene expression of renal cancer samples was performed using edgeR Bioconductor package using the classic edgeR design for comparing selected tumors to all nontumor tissues. We used Benjamini and Hochberg method of multiple testing correction with a 5% cutoff of FDR. We also filtered out genes with absolute log2FC values below 1 (genes with differential fold-changes below 2) (Supplementary Table 8).

### Identification of aberration hubs

We used one-tail Fisher's exact test as a statistical approach to evaluate enrichment of genomic or transcriptomic aberrations at the level of each subnetwork. Fisher's exact test was used to calculate probability of significance for the number of deregulated interacting partners of a protein by taking into account the total size as well as number of all deregulated proteins in the interactome. In this setup, we calculated p-values against the null hypothesis that interacting neighborhood of each protein shows a random enrichment in deregulated molecules. To correct for multiple testing, we used a permutation approach as discussed below. The reason for this is that the complex structure of the protein interaction network would create dependencies among the p-values.

### Multiple testing correction

In AbHAC, we performed Fisher's exact test on local neighborhood of each protein independently while complex interactions exist among the proteins. To estimate a false discovery rate based on the p-values, for 100 times we generate permuted networks and calculate the p-value for all of the proteins at each iteration. For this purpose, we swapped the label of the proteins that have similar number of interacting partners. Proteins with degrees >60 were placed in four equal size groups, with other groups consisting of proteins with exact same degrees, and labels were randomly swapped within each group. We defined the FDR as the median number of proteins passing significance threshold in the 100 permuted networks divided by the number of proteins passing the same significance threshold in the actual network. We then found the p-value cutoff corresponding to FDR of 5% by iterating through sorted p-values.

### Proteome and phosphoproteome data analysis

We obtained information about protein and phosphopeptide assembly and their relative abundance for TCGA breast cancer samples from clinical proteomic tumor analysis consortium (CPTAC; https://cptac-data-portal.georgetown.edu/cptac/s/S015). For each aberration hub, we examined if the protein or any of its phosphopeptides had a significantly different level in comparison of one subtype against other subtypes (t-test p-value < 0.05 & |fold change| > 0.5).

### Pathway analysis

We used ConsensusPathDB [50] for pathway enrichment analysis and used the significance cutoff of 0.05 for multiple test corrected p-values. Results of KEGG and PID from this software are provided as figures and tables in the manuscript.

### Cell culture

The established renal cancer cell lines A498 and 786-O were purchased from American Type Culture Collection (ATCC) (Rockville, MD, USA). The cell lines were cultured according to the recommendations of ATCC in the appropriate cell culture media and were incubated at 37°C in a humidified incubator with 5% CO2.

### siRNA transfection

The siRNA-mediated knockdown (KD) of SYK was carried out in 786-O and A498 cells. Briefly, two Silencer Pre-designed siRNA against SYK long isoform [29] with following sequences were used: SYK-L1 siRNA sequence: 5’-GUUCCCAUCCUGCGACUUGTT-3’ (sense); 5’-CAAGUCGCAGGAUGGGAACTT-3’ (antisense) and SYK-L2 siRNA sequence: 5’-GGUCAGCGGGUGGAAUAAUTT-3’ (sense); 5’-AUUAUUCCACCCGCUGACCTT-3’ (antisense). Silencer Negative Control siRNA (Ambion) was used as a control. Cancer cells were reverse transfected with siRNAs (final concentration of 35 nM) using Lipofectamine RNAiMAX Transfection Reagent (Invitrogen Corporation) according to the manufacturer's instructions.

### Western blot analysis

Protein extraction and western blotting from cell lines were performed as previously described [51]. Briefly, cells were washed with cold PBS/1mM Na_3_VO_4_/10mM NaF. Protein isolation was performed using a scraper in the presence of cold M-PER lysis buffer (Thermo Scientific, Rockford, USA) containing 1mM Na_3_VO_4_, 10mM NaF, anti-phosphatase and protease inhibitor. Protein was quantified using the BCA protein assay and was mixed with Laemmli Sample Buffer (Bio-Rad Laboratories, Mississauga, Ontario, Canada). After denaturing the lysate at 98°C for 6 min, 10 μg of each protein lysate was separated by 1DSDS-PAGE (10%) and, transferred to nitrocellulose membranes. After blocking for 1 hr with 5%(w/v) non-fat milk or BSA in TBST (50 mM Tris-HCl pH 7.4, 150 mM NaCl, 0.1% Tween 20), membranes were incubated with primary antibodies overnight at 4°C followed by secondary antibodies (HRP-conjugated anti- mouse IgG) for 1hr at room temperature. Signals were detected with ChemiDoc Touch Imaging System (Bio-Rad Laboratories, Mississauga, Ontario, Canada) using prime ECL plus (Sigma). The primary and secondary antibodies are as follows: Anti-mouse IgG HRP-linked (sc-2005), GAPDH (sc-365062) and SYK (sc-1240) from Santa Cruz Biotechnology. For the tissue analysis of SYK, extracts were prepared from frozen sections of matched normal and tumor (ccRCC) by addition of Laemelli buffer to 10 um sections (total ~1 cm^2^ surface area). After protein quantitation by a modified Bradford assay, 5 μg protein were loaded per lane of a Criterion TGX 8-16% gradient gel (Biorad) and the blotting procedure carried out essentially as above but with a 2 hour primary antibody incubation (1:20,000 dilution of anti-SYK). A Coomassie-stained parallel gel and densitometry was used to check equal loading together with Ponceau staining of the membrane to check for transfer.

### Colony-formation assay

Cells transfected with siRNA constructs were trypsinized and plated in a 6-well plate as single cells (1000 cells/well). Every 4 days, the growth medium was removed, the cells were washed with fresh medium once, and then fresh medium was added to the wells. Colony formation assays were followed for 8 days, then the medium was removed and the cells were washed with PBS. Washed cells were fixed for 10 min by adding 3.7% Paraformaldehyde (Alfa Aesar, Great Britain), and 0.05% (v/v) crystal violet (Acros, New Jersey, USA) was added to cells for 10 min in order to obtain visible colonies. Images were acquired using scanner Epson perfection V800 (Epson, Jakarta, Indonesia).

### Quantitative real-time PCR

Total RNA was extracted from cells using miRNeasy kit (Qiagen) according to the supplier protocols. 1μg RNA was reverse transcribed into complementary DNA (cDNA) using Transcriptor First Strand cDNA Synthesis Kit (Roche, Germany) following instructions provided by the manufacturer. Real-time PCR reactions were prepared using Lightcycler 480 SYBR green I master kit (Roche), and were run on a LightCycler 480 instrument (Roche) according to the manufacturer's recommendations. Triplicate PCR reactions were performed for each sample to ensure reliability. Expression of SYK-L mRNA was normalized to the expression of the housekeeping gene actin using the 2–[delta][delta]Ct method. The sequences of SYK and ACTIN primers were as follows:

ACTIN Forward, d (AGGCACCAGGGCGTGAT);

ACTIN Reverse, d (GCCCACATAGGAATCCTT CTGAC);

SYK-Long Forward, d (AGGGAAAGAAGTTCG ACACGCT);

SYK-Long Reverse, d (TTATTCCACCCGCTGAC CAAGT).

### Availability

AbHAC is available as a user friendly publicly available R package in github under GPL-3 license (https://github.com/mehrankr/AbHAC). Scripts for reproducing tables and figures of this manuscript are also made available (https://bitbucket.org/mkarimzadeh/abhac_suppcodes).

### Cell viability and caspase 3/7 activity

CellTiter-Glo and Caspase-Glo 3/7 Assay kits (Promega, Madison, WI) were used to assess cell viability and apoptosis induction 48h (apoptosis) or 96h (cell viability) after siRNA transfection as previously described [52].

### Cell cycle analysis

siRNA transfected cells were harvested after 48h by Accutase cell detachment solution (Sigma) and washed with cold PBS. Next we used cold Nicoletti buffer [53] for staining, and the DNA content of single nuclei was analyzed by BD FACSCanto II Flow Cytometry Analyzer Systems with collection of at least 10,000 events for each sample. The experiments were performed in triplicates.

## SUPPLEMENTARY MATERIALS FIGURES AND TABLES




