# Supplementary material for: Aberration hubs in protein interaction networks highlight actionable targets in cancer
Source: Oncotarget. 2018 May 18;9(38):25166–80. doi: 10.18632/oncotarget.25382 (PMC5982744; doi:10.18632/oncotarget.25382)
Supplement: Supplementary file 1 [file oncotarget-09-25166-s001.pdf]

Aberration hubs in protein interaction networks highlight actionable targets in cancer

SUPPLEMENTARY MATERIALS

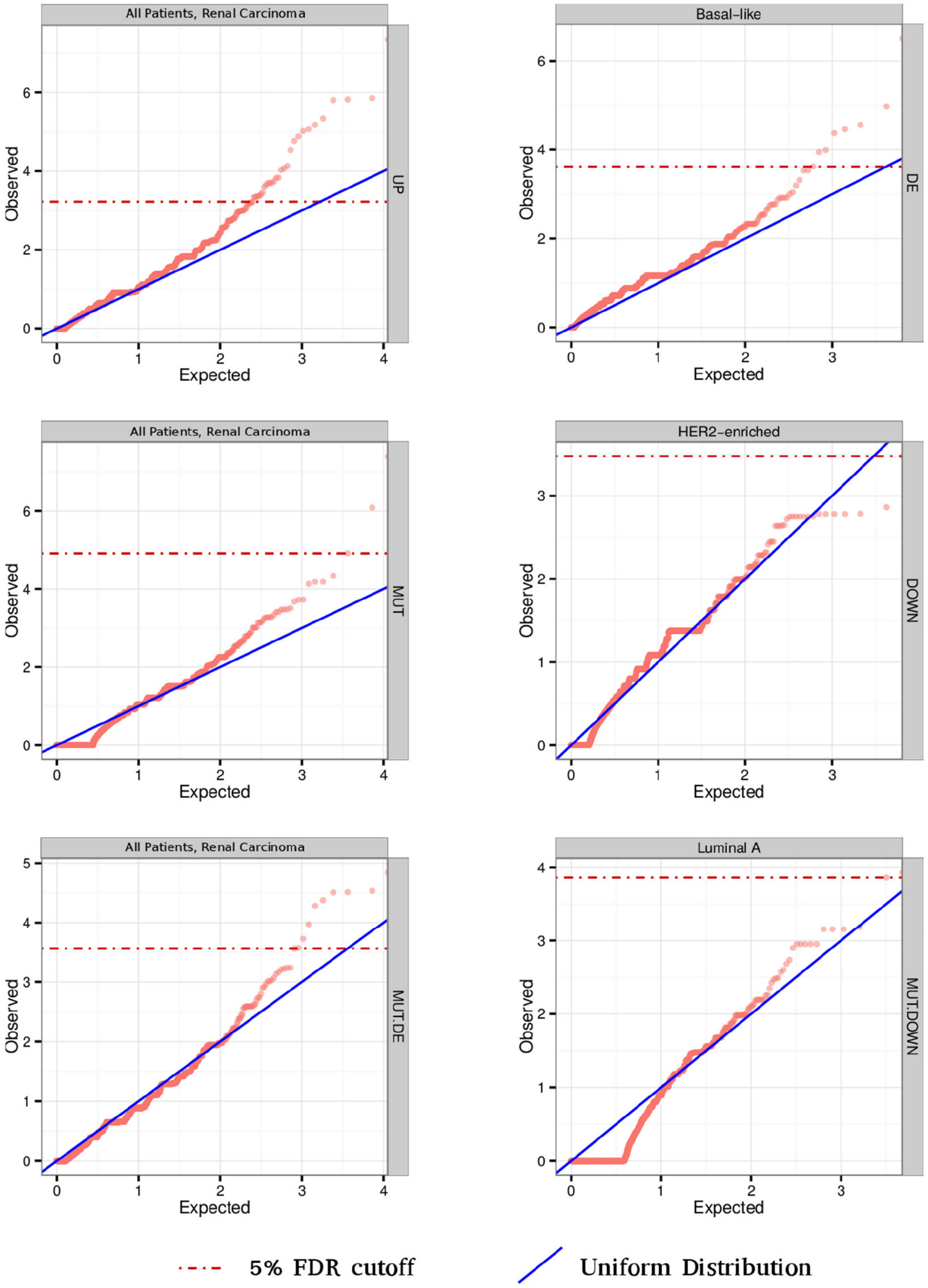

Supplementary Figure 1: QQ-plots for distribution of p-values obtained from AbHAC Fisher's exact test in breast and renal tumors. In some cases, such as aberration hubs enriched in downregulated genes of HER2-enriched breast cancers, no aberration hub passes the false discovery rate cutoff showed by the red dashed line.

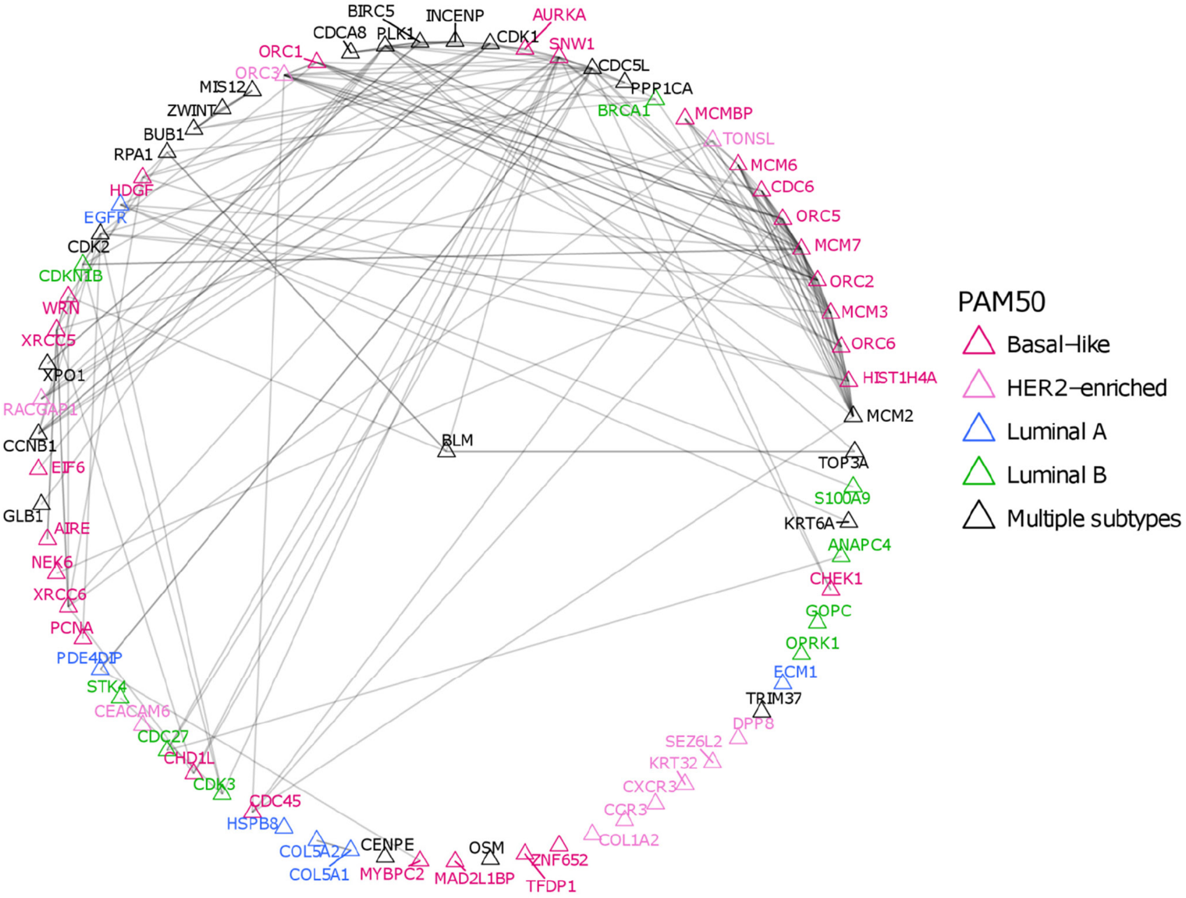

Supplementary Figure 2: Network of aberration hubs specific to PAM50 subtypes of breast cancer. We identified 74 aberration hubs by analyzing mutation and gene expression data of four PAM50 subtypes of breast cancer. Definition of aberration categories are provided in Table 1. Although identified independently, many of aberration hubs identified in basal-like tumors are connected together through interaction networks. Gray lines represent direct protein interaction and colour lines assist showing protein name. Network of protein interaction among the 74 aberration hubs. Colour of aberration hubs represents the corresponding PAM50 subtype. OSM is an aberration hub that was identified in basal-like tumors in aberration category of DOWN, and in Luminal A tumors in aberration category DE.

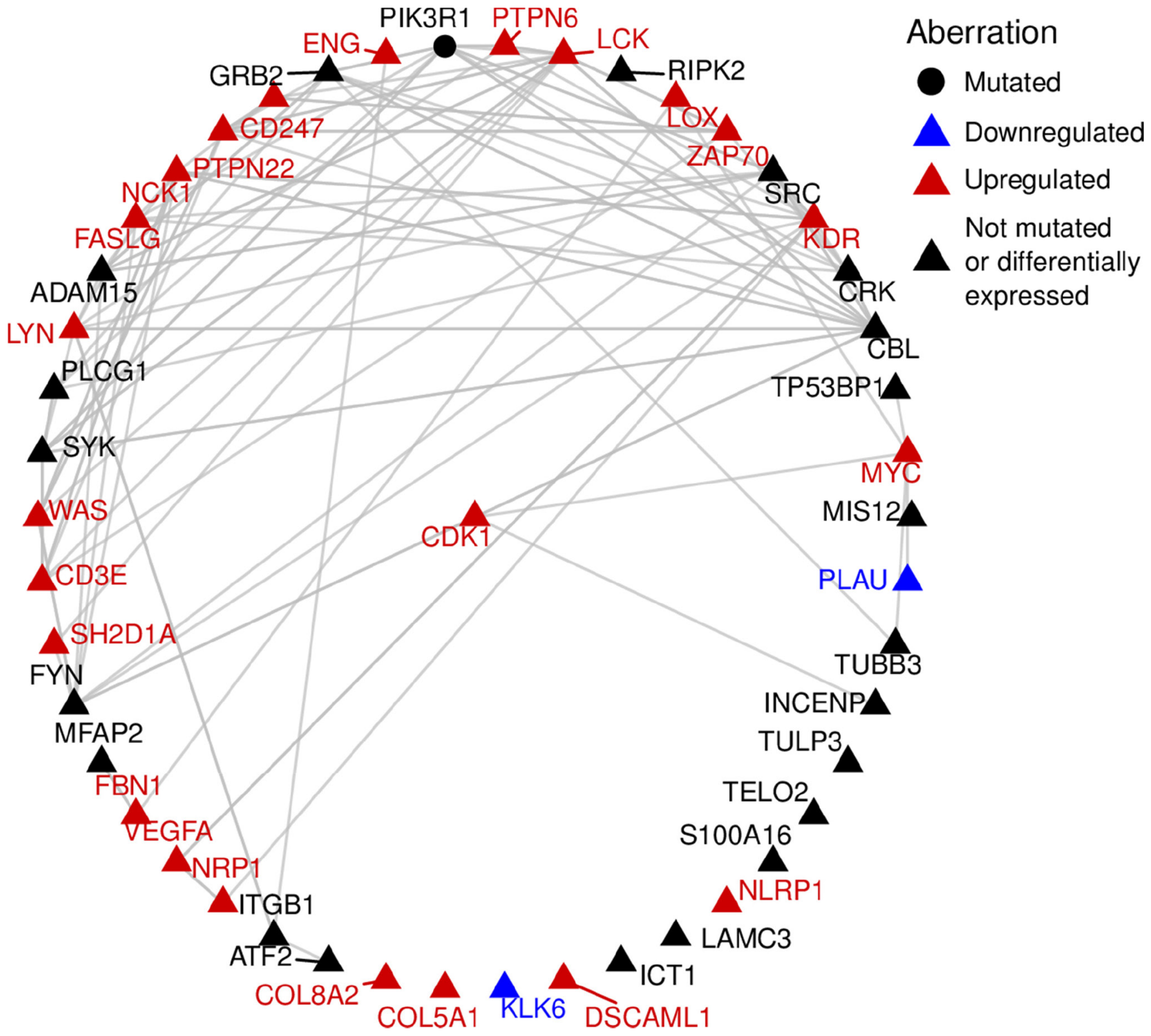

Supplementary Figure 3: Network of the direct protein interaction among aberration hubs identified in ccRCCs (FDR < 0.05; see Table 3 for the aberration categories). Color code represents the mRNA differential expression status of aberration hubs. Red and blue colors highlight aberration hubs that are upregulated or downregulated at mRNA level in tumors as compared to normal samples, respectively. PI3KR1 (circle) is the only aberration hub with somatic mutation. Black color aberration hubs do not show differential mRNA expression between tumor and normal samples.

a

Transcript	Transcript Length	Protein Length	Number of Exons	Abbreviation
ENST00000375754	5005	635aa	14	SYK-L1
ENST00000375746	4990	635aa	14	SYK-L2
ENST00000375751	4936	612aa	13	SYK-S1
ENST00000375747	4908	612aa	13	SYK-S2

b

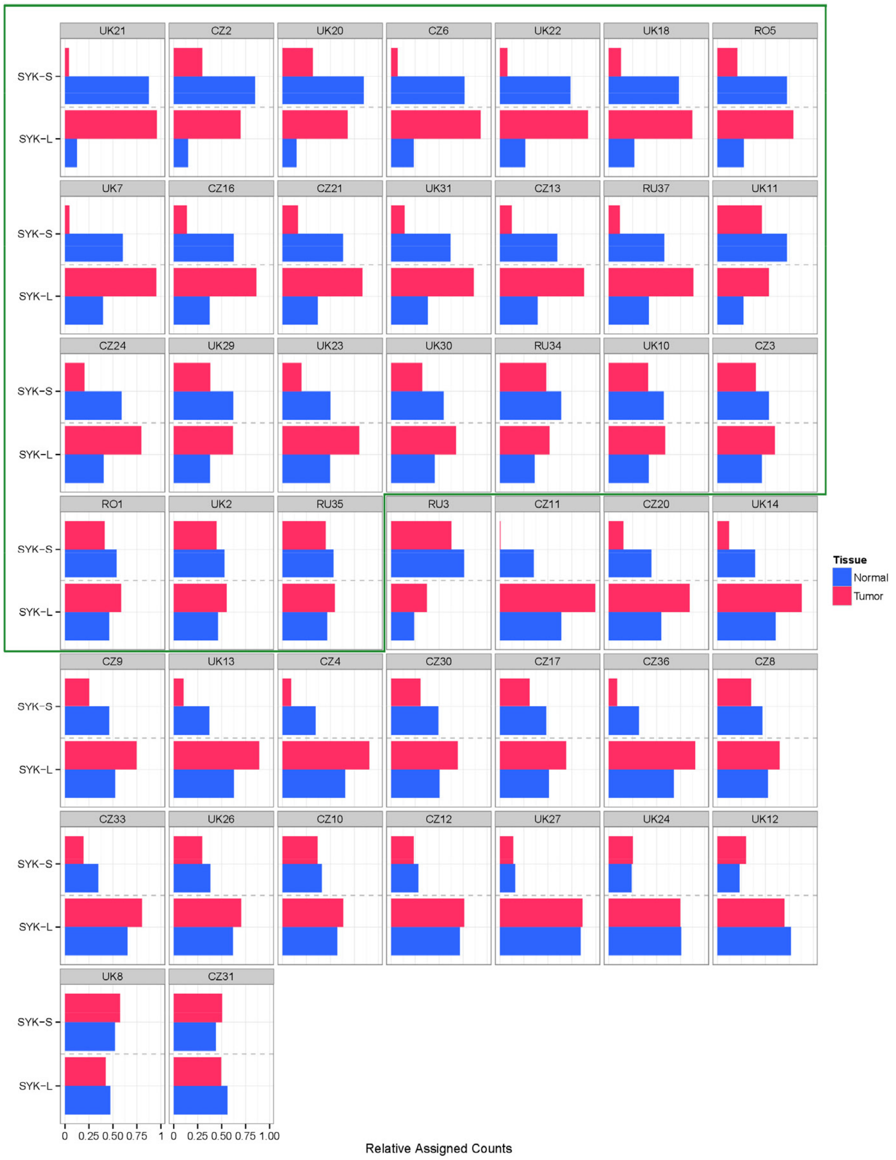

Supplementary Figure 4: SYK transcripts and their expression patterns in renal cancer and non-tumor samples. (a) There are 4 transcripts that give rise to 2 different protein products; the SYK long isoform and the SYK Short isoform. **(b)** Pooled relative abundance of transcripts with the same protein product is shown in tumor and patient-matched normal kidney tissue for 44 patients. Patient IDs are shown on top of each box. The green box includes patients with SYK-S as the major (most abundant) transcript in normal and SYK-L as the major transcript in tumor. Although in other patients there is no switch of major transcript between normal and tumor tissue, the relative abundance of SYK-L transcript shows an increase in tumor tissues.

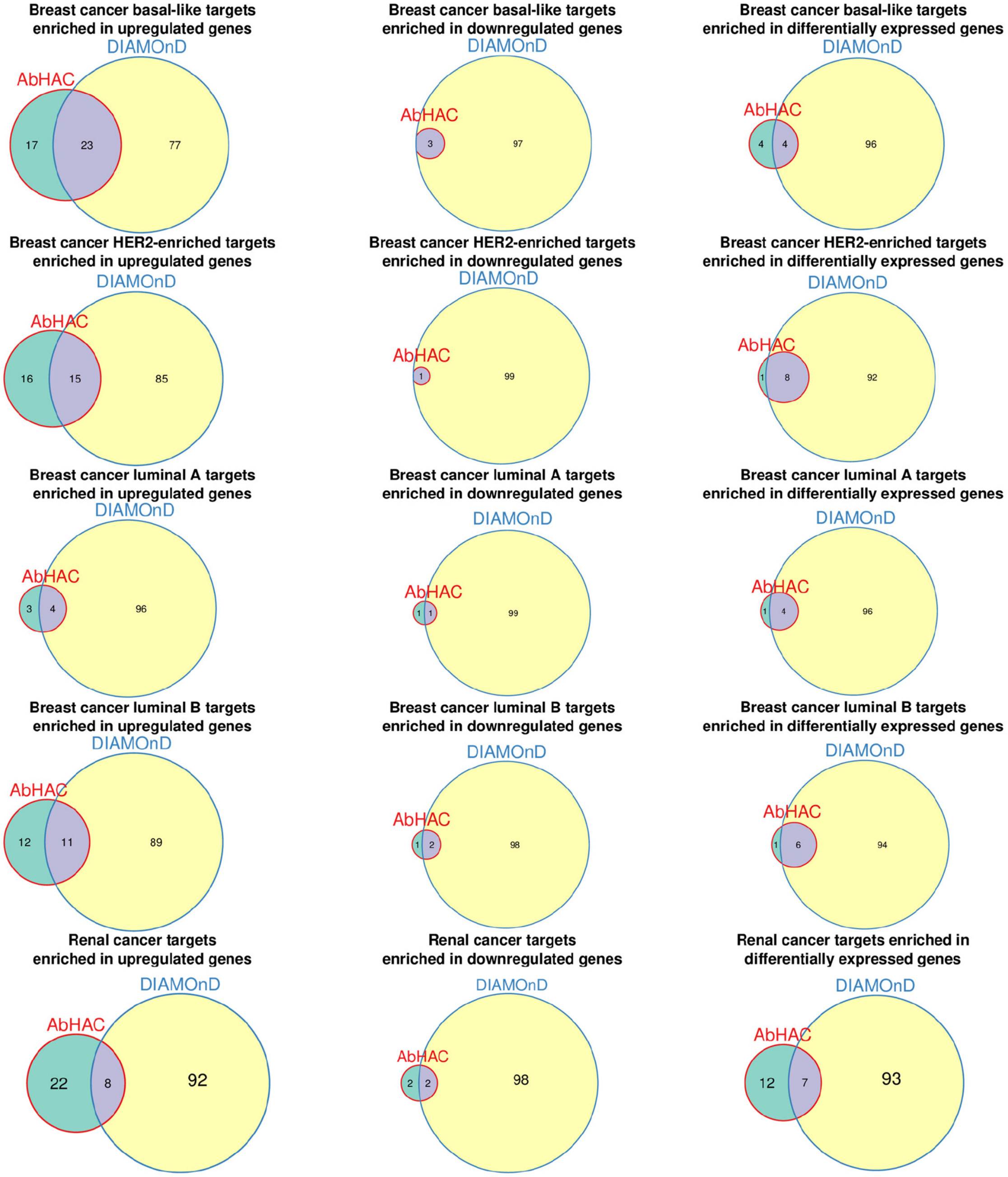

Supplementary Figure 5: Venn diagrams represent number of shared targets between AbHAC (FDR < 0.05) and DIAMoND (top 100 ranked targets) in a similar comparison for breast and renal carcinoma datasets.

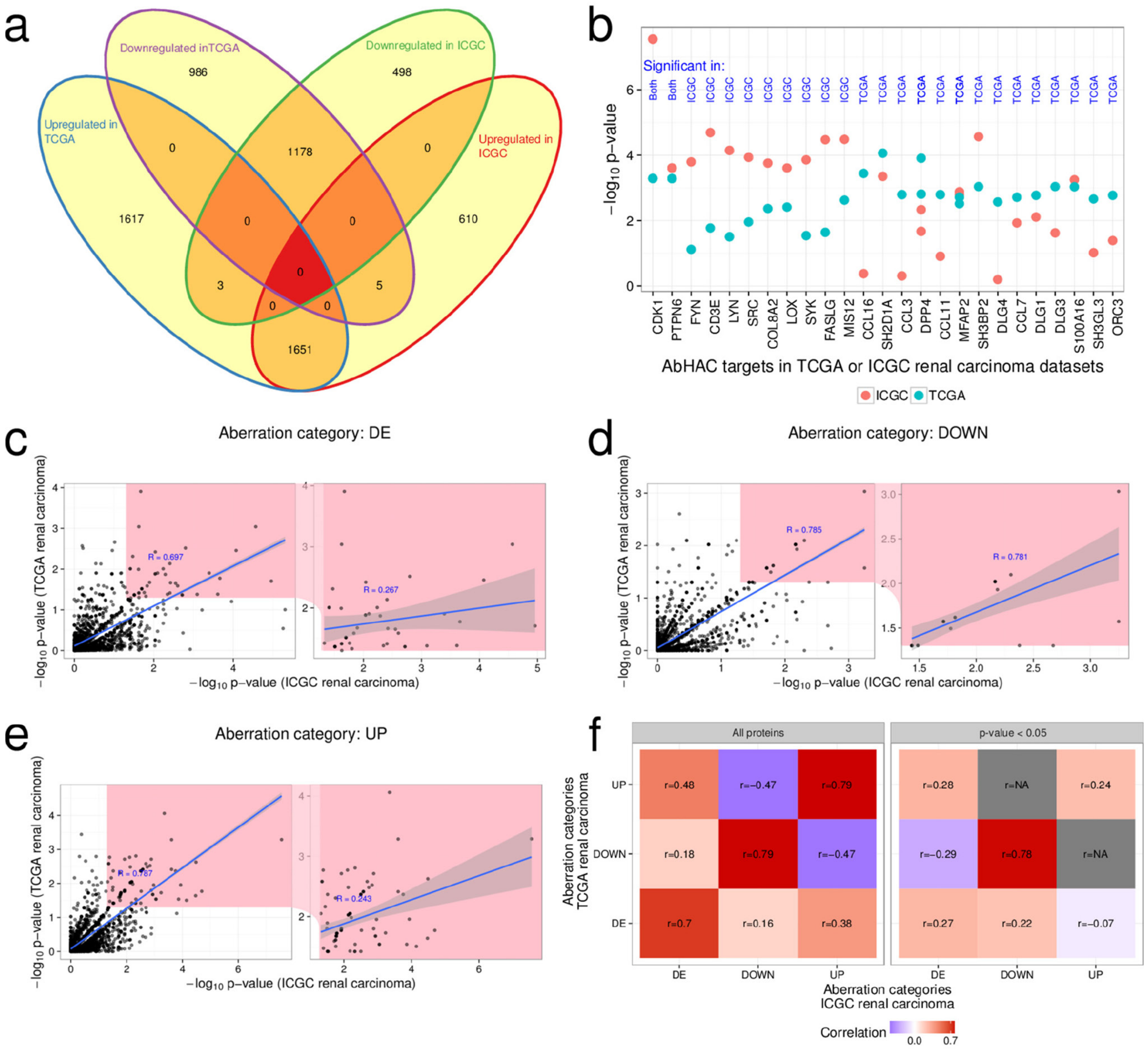

Supplementary Figure 6: (a) Venn diagram showing the number of upregulated and downregulated genes in ICGC renal carcinoma and TCGA renal carcinoma dataset tumors compared to non-tumor adjacent tissues. **(b)** AbHAC targets (Only in UP, DE, and DOWN aberration categories) that have a false discovery rate of less than 5\% in either ICGC renal carcinoma dataset, or TCGA renal carcinoma dataset, or both. Y axis shows not adjusted AbHAC p-values in the two datasets. **(c-e)** show correlation of AbHAC p-values in ICGC renal carcinoma and TCGA renal carcinoma datasets. **(f)** shows value of pearson correlation in comparison of various aberration categories between TCGA renal carcinoma dataset and ICGC renal carcinoma dataset.

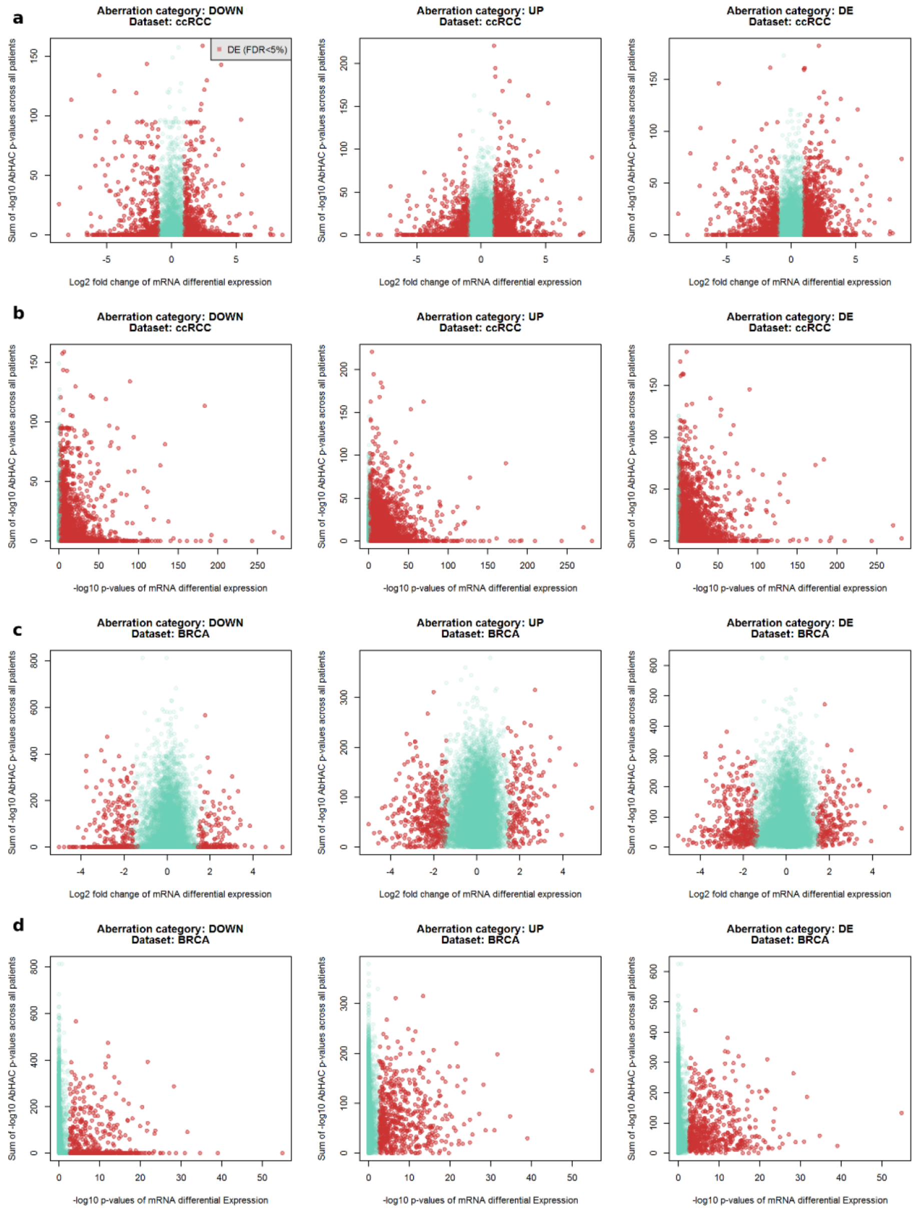

Supplementary Figure 7: Investigating deregulated neighborhood of an aberration hub and deregulation of the hub itself. Correlations between mRNA expression patterns of a gene, from gene expression analysis, and the status of deregulated neighborhood of its protein as analyzed by AbHAC were investigated. Log2 FC (tumor/normal) of mRNA expression of genes versus sum of patient-level AbHAC p-values (-log10 transformed) of the corresponding proteins across all patients for TCGA breast cancer **(a)** and CAGEKID renal cell carcinoma **(c)** datasets are plotted. Points of high values on Y axis correspond to proteins with a significantly deregulated neighborhood in a large number of patients. This analysis has been performed for three different aberration categories of deregulated neighborhood as mentioned on top of each plot. Red datapoints correspond to proteins whose mRNAs were identified as significantly differentially expressed between tumor and normal samples. **(b)** and **(d)** show similar analysis in breast and renal cancer datasets, respectively. However, this time p-values from differential gene expression were plotted against sum of AbHAC -log10 p-values for the corresponding proteins across all patients. These plots show that molecules with a neighborhood that is enriched in differentially expressed molecules are not necessarily differentially expressed themselves.

Supplementary Table 1: Mutated genes and instrinsic subtype (PAM50 group) in breast cancer

See Supplementary File 1

Supplementary Table 2: Diffrenetially expressed genes in all breast cancers compared to normal control samples (FDR<0.01)

See Supplementary File 1

Supplementary Table 3: Diffrenetially expressed genes in Basal-like breast cancers compared to normal controls samples (FDR<0.01)

See Supplementary File 1

Supplementary Table 4: Diffrenetially expressed genes in HER2-enriched breast cancers compared to normal control samples (FDR<0.01)

See Supplementary File 1

Supplementary Table 5: Diffrenetially expressed genes in Luminal A breast cancers compared to normal control samples (FDR<0.01)

See Supplementary File 1

Supplementary Table 6: Diffrenetially expressed genes in Luminal B breast cancers compared to normal control samples (FDR<0.01)

See Supplementary File 1

Supplementary Table 7: Mutated genes in clear cell renal cell carcinomas

See Supplementary File 1

Supplementary Table 8: Diffrenetially expressed genes in clear cell renal cell carcinomas compared to normal control samples (FDR<0.01)

See Supplementary File 1

Supplementary Table 9: Aberration hubs identifed in intrinsic subtypes of breast cancers (FDR<0.05)

See Supplementary File 1

Supplementary Table 10a: ConsensusPathDB pathway analysis for aberration hubs specific to Basal-like breast tumors

See Supplementary File 1

Supplementary Table 10b: ConsensusPathDB pathway analysis for aberration hubs specific to HER2 breast tumors

See Supplementary File 1

Supplementary Table 10c: ConsensusPathDB pathway analysis for aberration hubs specific to Luminal breast tumors

See Supplementary File 1

Supplementray Table 11: Breast cancer AbHAC targets without damaging somatic mutations or abnormal expression at mRNA level that showed significant differences in phosphopeptide expression in the same PAM50 subtype found by AbHAC as compared to tumors with other subtypes

See Supplementary File 1

Supplementary Table 12: Aberration hubs identifed in renal cell carcinoma (FDR<0.05)

See Supplementary File 1

Supplementary Table 13a:Targets identified by AbHAC or DIAMOnD in renal carcinoma datasets

See Supplementary File 1

Supplementary Table 13b: Targets identified by AbHAC or DIAMOnD in breast carcinoma datasets

See Supplementary File 1
